# Rational Design of Smart Hydrogels for Biomedical Applications

**DOI:** 10.3389/fchem.2020.615665

**Published:** 2021-02-04

**Authors:** Yanyu Zhang, Yishun Huang

**Affiliations:** ^1^ Institute of Analytical Technology and Smart Instruments, Xiamen Huaxia University, Xiamen, China; ^2^ Engineering Research Center of Fujian Province, Xiamen Huaxia University, Xiamen, China

**Keywords:** hydrogel, responsibility, biomedical, natural polymer, synthetic polymer

## Abstract

Hydrogels are polymeric three-dimensional network structures with high water content. Due to their superior biocompatibility and low toxicity, hydrogels play a significant role in the biomedical fields. Hydrogels are categorized by the composition from natural polymers to synthetic polymers. To meet the complicated situation in the biomedical applications, suitable host–guest supramolecular interactions are rationally selected. This review will have an introduction of hydrogel classification based on the formulation molecules, and then a discussion over the rational design of the intelligent hydrogel to the environmental stimuli such as temperature, irradiation, pH, and targeted biomolecules. Further, the applications of rationally designed smart hydrogels in the biomedical field will be presented, such as tissue repair, drug delivery, and cancer therapy. Finally, the perspectives and the challenges of smart hydrogels will be outlined.

## Introduction

Hydrogels are cross-linked three-dimensional hydrophilic networks with the ability to maintain large amounts of water with tunable biocompatibility, biodegradability, acute environmental sensing, and mechanical properties (Huang et al., 2017; Palmese et al., 2019).

Hydrogels can be designed by the incorporation of natural or synthetic polymers through physical or covalent cross-linking. Their properties can be adjusted to meet the diverse applicable demands by the variation of hydrophilic and hydrophobic proportion or the addition of active recognition motif (Ferreira et al., 2018; Kasiński et al., 2020). Hydrogels are considered to be the most prospective alternative materials for soft tissue due to their exceptional mechanical properties (Li and Su, 2018; Wang W. et al., 2018). The excellent properties endow hydrogels as superb materials for local drug delivery or external stimuli sensing which make them more functional in biomedical applications than traditional chemical sensors.

**FIGURE 1:**
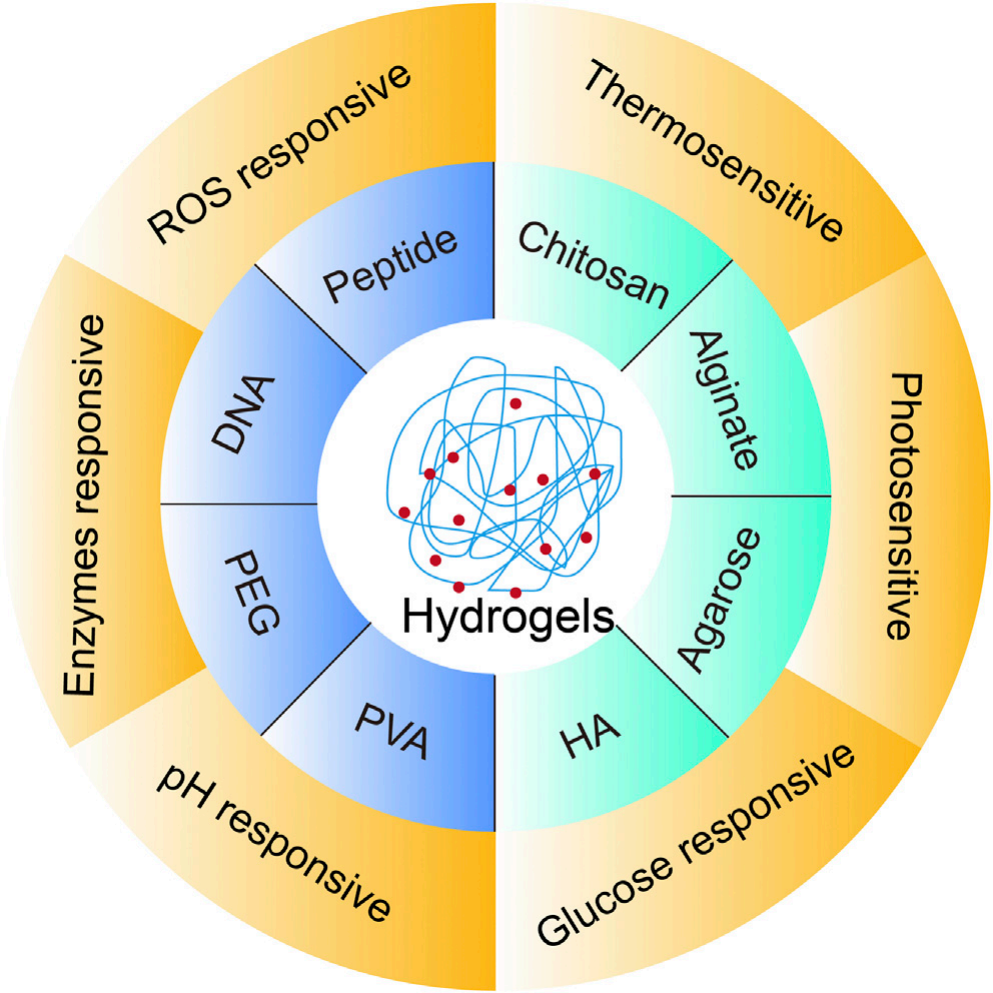
Schematic diagrams showing the rational design of smart hydrogels.

As shown in Figure 1, the mini review will have an introduction of classification by hydrogel-forming molecules that present the structural basis for intelligent hydrogel design. There are also other classifying standards exploited by other reviews, such as cross-linking type (Hu et al., 2019; Sharma and Tiwari, 2020). Meanwhile, the discussion on the molecular pros and cons in designing is included. Then, the rational design of intelligent hydrogels to the environmental stimuli such as temperature, irradiation, pH, and targeted biomolecules is presented in the biomedical applications, such as tissue repair, drug delivery, and cancer therapy. Finally, the perspectives and the challenges of smart hydrogel design will be outlined.

## Classification of Hydrogels

Hydrogels can be divided in two categories by the forming molecule types, natural polymer and synthetic polymer. Commonly, naturally derived hydrogels including cellulose (Isobe et al., 2018), chitosan (Ouyang et al., 2018), alginate (Hao et al., 2017), and agarose (Bilal et al., 2019) which are common in the natural environment. They keep their biochemical and biocompatible properties with the host tissue (Pena et al., 2018), although with relatively weak mechanical strength, difficulty in reproducing accurate formulation and drug loading, and potential immunogenic risks. Synthetic polymers are man-made polymers derived from polymerized of monomer. Hydrogels made of synthetic polymers like poly(ethylene glycol) (PEG) (Cruz-Acuna et al., 2018), poly(vinyl alcohol) (PVA) (Zhang Y. et al., 2019), and synthetic biopolymers including peptide (Mondal et al., 2020) and DNA (Gačanin et al., 2020) possess high water absorption capacity, well-defined structure, wide varieties of raw chemical resources, and intelligent reply to different stimuli. Herein, the inherent nature of hydrogel-forming molecules is focused on to discuss the structural basis for hydrogel forming and corresponding hydrogel application.

### Natural Polymer

#### Chitosan

Chitin, a natural mucopolysaccharide, is one of the most plentiful polysaccharides on earth, ranking second to cellulose (Tao et al., 2020). Chitosan is the major derivate of chitin obtained from the deacetylation of chitin. It is reported that hydrogels made of chitosan have wound-healing, antitumor, and hemostatic properties (Antony et al., 2019; Peers et al., 2020), due to its negligible toxicity and good biodegradability. However, most high molecular weight chitosan is insoluble in water caused by strong intramolecular hydrogen bonding, which hinders the applications. To overcome the limitation, functional addition of hydrophilic groups on the amine and hydroxyl group is a convenient approach to tune the solubility (Alizadeh et al., 2019; Li N. et al., 2019). Sheng et al. (2020) synthesized a novel bioactive photothermal hydrogel based on N, O-carboxymethyl chitosan incorporated fayalite (Fe2SiO4), which could release bioactive ions with mild heating function to in wound area to enhance angiogenesis and chronic wound healing.

#### Alginate

Alginate is a natural, anionic linear polysaccharide composed by two types of rudimentary copolymers, 1,4-β-d-mannuronic acid (M) and 1,4-α-l-guluronic acid (G) which are organized in a block (-M-M-M- or -G-G-G-) or alternating (-M-GM-G-) configuration. Alginate is able to chelate with divalent cations to form physical hydrogels such as Ca2+ and Ba2+ with G block which prompts the generation of ionic bridge between nearby polymer chains (Sheng et al., 2020). The proportion of the repeated units and the length of the chain will change the function of polymer. The exceptional features of alginate, such as good biocompatibility, biodegradability, and low toxicity, have endowed it with outstanding performance in tissue engineering, drug delivery, and 3D bioprinting (Rastogi and Kandasubramanian, 2019; Zhao et al., 2020b; Axpe and Oyen, 2016). Although the inadequate mechanical stability, poor cells adhesiveness and relatively slow degradation *in vivo* hinder the further *in vivo* application. Garcia-Astrain and Averous (2018) developed a novel alginate-based hydrogel *via* Diels–Alder click reaction by furan–alginate polymer and bifunctional cross-linkers with different swelling and degradation behaviors. The alginate-based hydrogel could be wonderful candidates to apply in biomedical fields.

#### Agarose

Agarose, a neutral linear polysaccharide derived from marine algae, is composed of d-galactose and 3,6-anhydro-l-galactose linked disaccharide units. The gelling mechanism of agarose depends on the aggregation of double helices formed by intermolecular hydrogen bonds (Cambria et al., 2020). The feature of biocompatibility, biodegradability, and strong gelling upon gentle conditions renders agarose biopolymer extremely appealing as a promising biomaterial in antiviral treatment, cancer therapy, and colorimetric biosensing (Kim et al., 2020; Lima-Sousa et al., 2020; Zhao L. et al., 2020). The hindrance in biomedical applications lies in the low emulsifying activity and low cell attachment ability (Kumar et al., 2018; Xiao et al., 2020). However, the property of agarose hydrogel can be readily modified by incorporation with other biopolymers. Specially, mixing with chitosan can significantly ameliorate hydrogel properties, such as mechanical and cell-adhesive ability (Bilal et al., 2019).

#### Hyaluronic Acid

Hyaluronic acid (HA) with linear structure and molecular weight ranging from 6,500 to 10,900 kDa is a component of all connective tissue (Jensen et al., 2020). It is an anionic and non-sulfated glycosaminoglycan composed of alternating units of a repeating disaccharide, composed by d-glucuronic acid and N-acetyl-d-glucosamine (Ahmadian et al., 2019; Murthy et al., 2019). HA is abundant in proliferative tissues during embryogenesis, regeneration, and carcinogenesis as it is a suitable environment for cell migration and proliferation (Sánchez et al., 2020). HA-based scaffolds have been prepared in the form of hydrogels (Silva Garcia et al., 2019), meshes (Chen et al., 2020), and sponges (Liu J. Y. et al., 2018). HA and its derivatives are also widely applied to dermal fillers (Bukhari et al., 2018), wound dressings (Graca et al., 2020), drug delivery (Huang and Huang, 2018), and tissue engineering (Pandit et al., 2019) due to its extraordinary water-retaining ability, biocompatibility, biodegradability, hydrophilicity, and non-immunogenicity (Choi et al., 2019). However, due to the molecular structure and molecular weight, HA can form soluble molecular networks but not just physical form. Thus, to generate HA hydrogels, chemical modifications, covalent cross-linking, and gelling agents are recommended (Trombino et al., 2019). To improve the stability and biological half-life, HA is modified with hydrophobic groups to develop with other superior properties (Huang and Huang, 2018). Various modification methods like esterification and auto reaction with divinyl sulfone are utilized to design the properties of HA (Bermejo-Velasco et al., 2018; Zhang M. et al., 2019). For example, Kwon *et al. *(Kwon et al., 2019) modified HA with different chemical groups to quantify its binding affinity to CD44, which was applied to explore the downstream effects of modified HA hydrogel on mesenchymal stromal cell chondrogenesis.

#### Other Natural Polymers

Other naturally derived polymers such as gelatin, collagen, dextran, cyclodextrin, and fibrin also have been inclusively applied at tissue repair (Klotz et al., 2016), wound healing (Ying et al., 2019), and drugs delivery (Wu et al., 2014; Liu et al., 2016; Liu G. et al., 2018). Among these natural polymers, gelatin is a biodegradable protein, mainly derivate of porcine or bovine (Gibbs et al., 2016), with good biocompatibility, biodegradability, plasticity, and adhesiveness. But the high hygroscopicity and poor mechanical properties restrict their applications (Wang et al., 2019). Collagen is one of the extracellular matrix proteins. Their osteoinductive is especially suitable for cartilage repair, but poor strength and fracture toughness become a barrier for their applications (Lu et al., 2019). In regard to gelatin and collagen, blending with other polymers or modified by functional groups was commonly adopted to improve their applications. Dextran is a linear α-1, 6 linked d-glucopyranose residues with excellent biocompatibility and chemical functionality (Du et al., 2019). Fibrin is generated by the polymerization of fibrinogen to form fibrillar scaffold. When tissue injury happened, fibrin clot was formed by fibrinogen and platelets. As for dextran and fibrin, they have been approved by the FDA and widely employed in biomedical applications, such as anti-inflammatory (Lee et al., 2018; Tanaka et al., 2019) and tissue regeneration (Marcinczyk et al., 2017) drug delivery.

### Synthetic Polymers

#### PEG

PEG is a water-soluble, non-immunogenic, and biocompatible molecule, which is nontoxic, inert, and suitable for use in medical products. PEG has linear and branched structures with two or more terminal hydroxyl groups. Those two hydroxyl groups can be further functioned with other groups, providing versatility for hydrogel preparation or biomolecule conjugation (D’Souza and Shegokar, 2016). Their excellent properties such as photopolymerization, adjustability, and controllability make PEG hydrogels a promising tool for drug delivery system (Zhu, 2010). Recently, PEG hydrogel serves as versatile biocompatible scaffolds with tunable stiffness precisely mimicking physiological and pathological microenvironments (Crocini et al., 2020). However, due to the bio-inert nature, PEG hydrogels cannot supply an ideal circumstance for sustaining cell adhesion and tissue formation alone (Lin and Anseth, 2009). Nam et al. (2019) created hydrogels through modification of PEG to alginate. This study showed that the total amount of PEG grafting onto alginate could regulate stress relaxation and would be a novel approach to tune stress relaxation in alginate hydrogels.

#### PVA

PVA, a synthetic hydrophilic polyhydroxyl polymer, has been accepted by the FDA as a biocompatible and nonantigenic compound (Yu F. et al., 2018). The varying molecular weight of PVA makes it versatile in polymer properties such as crystallizability, adhesion, and mechanical strength. Due to the ease of modification and favorable mechanical property, PVA has been largely employed in tissues such as the bone (Cheng et al., 2020), heart (Wu et al., 2018), nerve (Ribeiro et al., 2017), and vascular network (Li Z.J. et al., 2019). Especially, the viscoelastic properties are close to articulatory and meniscal cartilage, prompting them notably appealing biomaterials for applying in tissue engineering. However, the insufficient elasticity, rigid structure (Kamoun et al., 2015), and calcification in biological fluids for longer time (Hill et al., 2011) limited their applications. Thus, scientists are focusing on developing novel feature of PVA by mixing with other polymers, cross-linking, or grafting (Kamoun et al., 2017). In order to obtain superior PVA mechanical properties, various approaches have been attempted to fabricate hydrogels by regulating the physical, non-covalent cross-links, such as blending with gelatin, chitosan, and PEG (Charron et al., 2019). For example, Golafshan et al. (2017) design a new PVA–alginate hydrogel, which could supply with damp surroundings and be beneficial for accelerating wound healing.

#### Peptide

Peptides are composed of amino acids that are suitable synthetic polymer for the design of therapeutics and biomaterials (Sis and Webber, 2019). Due to the designated structure and chemical feature, peptides can be programed from secondary structure motifs to superior structure at nanoscale (Singh et al., 2017). The superb biodegradability, biocompatibility, bioactivity, and responsiveness make them potential in biomedical applications (Wang et al., 2017; Waduthanthri et al., 2019). Peptide self-assembly is usually driven by its amphiphilic characteristics to form peptide hydrogel. The assembly process could be reversed by external trigger, such as pH (Wiedman et al., 2017), concentration (Lim et al., 2017), and temperature change (Wang Y. et al., 2018; Matt et al., 2019). However, the mechanical properties of self-assembling peptide hydrogel are often weak (De Leon Rodriguez et al., 2016) because the utilized synthetic peptides are usually short peptides with less than 30 amino acids (Radvar and Azevedo, 2019). To improve the mechanical property, one strategy is to combine peptides with other polymeric networks, through covalently linking peptides to polymers, or by non-covalent interactions between peptides and polymers (Kopecek and Yang, 2012). For example, Clarke et al. (2017) prepared hybrid hydrogels composing of a poly (γ-glutamic acid) polymer and self-assembling β-sheet peptides by physically cross-linking with superior strain stability. In this work, they tuned the mechanical properties of the hydrogels by changing the β-sheet peptide grafted density and concentration to obtain hydrogels analogous to soft tissue.

#### DNA

Synthetic DNA, originated as a genetic molecule, possesses a distinct and fascinating characteristic, such as accurate base-paring recognition capability, sequence-dependent designability, and tunable multi-functionality (Zhou et al., 2020). These super properties offer exquisite platform for the forming functional hydrogels. DNA can be self-replicated to be long strands to cross-link each other to form RCA hydrogel (Geng et al., 2018). These designs make advantages of the DNA’s merits can endow the formed hydrogel with high designability and precise controllability (Shahbazi et al., 2018). The presence of functional DNA makes DNA hydrogel a good platform to sensing the external stimuli (Ma et al., 2017; Khajouei et al., 2020). However, because of multiple drugs or signal molecules are small molecules, DNA hydrogel networks show high permeability toward them. Besides, hydrophobic drugs add additional barriers in drug containing by DNA hydrogels because of the low encapsulating efficiency. Therefore, it is still a challenge to apply responsive DNA hydrogel in biomedical drug delivery (Lyu et al., 2018).

#### Other Synthetic Polymers

Apart from what we have mentioned above, there still exist other synthetically derived polymers (Wu et al., 2012; Pang et al., 2014; Zhang et al., 2018; Huang et al., 2019) used for hydrogels forming, such as poloxamers (like poloxamer 407(F127), poloxamer188(F-68)), and poly (N-isopropylacrylamide) (PNIPAM). Poloxamers are amphiphilic tri-block copolymers of poly(ethylene oxide) (PEO)–poly(propylene oxide) (PPO)-PEO. Poloxamers are extensively used in the drug delivery system and tissue regeneration scaffolders due to their good biocompatibility and solubility, low cytotoxicity, and superb rheological behavior around body temperature. Poloxamers form hydrogels above the lower critical solution temperature (LCST) while remaining solution when below it (Russo and Villa, 2019). PNIPAM with amide and propyl moieties made sol–gel transformation at about 32°C. PNIPAM forms hydrogels above 32°C driven by their hydrophobic interaction of propyl groups (Haq et al., 2017). They are widely applied in tissue engineering (Han et al., 2016) and drug delivery (Cao et al., 2019). F127 and PNIPAM are regularly used for thermo-responsive polymer. But their applications are limited by fast dissolution in aqueous solution for F127 and low bioactivity for PNIPAM (Lin et al., 2019; Yap and Yang, 2020). Therefore, F127 was modified with carboxymethyl hexanoyl chitosan to retard the dissolution of F127 (Yap and Yang, 2020). And PNIPAM is usually mixed or copolymerized with natural derived polymers to adjust its properties (Atoufi et al., 2019).

### Multipolymer

Multipolymer hydrogels are mainly involved interpenetrating polymer network (IPN) and semi-IPN (Ahmed, 2015). IPN is made of two independent interlaced polymer networks. Semi-IPN consists of at least one hydrogel network with one additional linear or branched polymer. Comparing with conventional homopolymer/copolymer hydrogels, multifunctional and multipolymer hydrogels are more widely employed in biomedical and pharmaceutical applications because of the several advantages, enhanced physical and mechanical properties, tunability, and targeting (Matricardi et al., 2013; Pacelli et al., 2018). Polysaccharides such as alginate, HA, and chitosan and proteins including gelatin and collagen are widely used to forming IPN or semi-IPN hydrogels. For example, *Park et al. (2019)* fabricated a hydrogel based on IPN by integrating gelatin with silk fibroin. Here, gelatin supplied for cell adhesion and proliferation with superior bioactivity and silk fibroin provides with excellent mechanical properties and biocompatibility. The resultant hydrogel showed more improved mechanical properties than one component alone. They would be promising in biomaterial applications. *Das et al. (2019)* developed a semi-IPN hydrogel with improved mechanical properties by cross-linking alginate with synthetic polymer *via* free radical polymerization. It is a potential application for drug delivery vehicle.

## Smart Hydrogel in Biomedical Application

By far, hydrogels can be formulated with various polymers to endow them diverse functionality in biomedical applications. Based on the responsive stimuli, smart hydrogels could be divided into physical, chemical, and biochemical responsive hydrogels. They are extensively applied in biomedical fields, including therapeutic delivery (Liang Y. et al., 2019), contact lenses (Alvarez-Rivera et al., 2018), corneal prosthesis (Koivusalo et al., 2019), wound healing (Zhang et al., 2020), bone regeneration (Bao et al., 2020), and tissue engineering (Kaiser et al., 2019; Tresoldi et al., 2019; George et al., 2020; Wang et al., 2020).

### Physical Responsive Hydrogels

Precisely, rational design for smart hydrogel in biomedical application is needed according to the inherent property of hydrogel and the molecular constructure, to meet the detailed and trivial demand of the practical biomedical applications.

Generally, physical stimuli such as temperature, light, and electricity can be easily modified, which make it the prioritized option for biomedical applications (Saludas et al., 2017).

#### Thermosensitive Hydrogels

Thermosensitive hydrogel is the most extensively evaluated and conventional type of stimuli-responsive gel system. They are defined by their capability to swell or shrink with the surrounding temperature changing (Ullah et al., 2015). The volume change is based on the proportion of hydrophobic and hydrophilic groups in the hydrogel-forming molecules. As in the water content hydrogel, the cross-linking force including the hydrophobic interaction and hydrogen bonding will vary as the temperature changes (Fan et al., 2019). Diverse biomaterials such as chitosan, agarose, and HA (Xu X. et al., 2019; Makvandi et al., 2020) are evolved and used for thermosensitive polymers to design smart hydrogels in the past.

There are diverse applications for thermosensitive hydrogels, including bacterial infected wound healing (Yan et al., 2019), tumor treatment (Jia et al., 2020; Qin et al., 2020; Yuan et al., 2020), and tissue regeneration (Zhao C. et al., 2020). Recently, Almoshari et al. (2020) synthesized an intelligent hydrogel by blending pyrophosphorylated Pluronic F127 (F127-PPi) with regular F127 to contain the hydrophobic glycogen synthase kinase 3 beta inhibitor, which could provide functions of inflammation modulating and osteoanagenesis. The solubility of inhibitor was improved by the amphiphilic F127 polymer at room temperature which makes it suitable for drug loading. Nevertheless, F127 transforms into a hydrogel state under physiological temperature with sustaining release behavior for localized therapeutics.

LCST based coil-to-globule transition of the crosslinker is the driven force to achieve thermosensitive sol–gel transformation (Graham et al., 2019). LCST of the crosslinker is determined by types of substituted hydrophobic groups and their molecular weight (Dai et al., 2019). Polymers, such as PNIPAM family, poly(d,l-lactide)–poly(ethylene glycol)- (PLEL) and polysaccharides, endow hydrogels with a unique feature of thermosensitive. By variation of end groups of NIPAM, the LCST can be tuned in the range of 32.8°C–45.3°C (Xia et al., 2006). Below LCST, the polymers are soluble. Above LCST, they become insoluble and result in gel forming. Zheng et al. (2020) employed catechol-modified quaternized chitosan, combined with PLEL to fabricate an injectable thermosensitive hydrogel with antibacterial and tissue adhesive properties. The PLEL polymer was in a random coil configuration when below its LCST. When the temperature reach above LCST, PLEL polymers aggregated together to form micelles with the hydrophilic PEG outside and hydrophobic poly (d, l-lactide) inside, respectively. The subsequent entanglement of PEG fragment causes the gelation of PLEL solution which in turn enhances the tissue adhesion at physiological temperature. The animals experiment revealed that the thermosensitive hydrogel could effectively close the broken skin and conspicuously facilitate wound healing.

#### Photosensitive Hydrogels

Photopolymerizable hydrogels attract considerable interest in drug delivery and tissue engineering fields because of their ability to be remotely administered in the formation of hydrogel by *in-situ* photopolymerization. The light stimulus, commonly UV or near-infrared light, can be imposed instantly and delivered with space-time accuracy, allowing overall control of the hydrogel formation with tunable properties (Meng et al., 2019; Joshi, 2021). When the hydrogel encapsulates the photosensitive materials, according to the photothermal or photodynamic properties, the hydrogels can be divided into two types. One is the addition of the photothermal material into the hydrogels which converts the light energy into heat energy (Jia et al., 2020; Zhao et al., 2020a). The other is the insertion of photodynamic moieties into the hydrogel structure which can support controllable photodynamic therapy (Chang et al., 2019). When the network of hydrogel is composed by photosensitive molecules (Wang et al., 2014; Fedele et al., 2018), including azobenzene, spiropyran, and *o*-naphthoquinone, after the light irradiation, molecular alteration will bring in hydrogel network changes. The ruthenium, nitrophenyl, and coumarin compounds, in which there are photo-cleavable groups, can be bonded to the hydrophobic end of the hydrogel crosslinker or scaffold. The ester group is broken to release the hydrophobic end under the light irradiation which will turn the gel–sol change or other property variations (Grim et al., 2015; Yao et al., 2018).

Photosensitive hydrogels are extensively applied in cell culture and 3D tumor microenvironment studies (Dong et al., 2018). Through optical fiber localized irradiation, Cai et al. (2019) designed a 3D photosensitive hydrogel in the nerve guidance conduit by utilization of phenyl azide to find that the photosensitive hydrogels formed inside maintain the collagen locally and have a better cell adhesion and survival rate. Expectantly, the system would be a promising hydrogel scaffold for spinal cord injury repair.

#### Shape Memory Hydrogels

Shape memory hydrogels (SMHs) possess the properties of shape memory transformation from temporary shape to eternal shape upon exposure to the stimuli, which endow them great potential in tissue engineering and drug delivery (Korde and Kandasubramanian, 2020; Liang et al., 2019a; Löwenberg et al., 2017). The SMHs have at least two types of hydrogel networks. One is the permanent hydrogel network to maintain the eternal shape. The other one is the transient network to turn the shape into a temporary shape and subsequent response to environmental stimuli to reverse. Traditional SMHs are evolved from the shape memory polymers which mainly contain the hydrophobic moieties to render thermo-responsive behavior (Osada and Matsuda, 1995; Behl et al., 2013). When altering the transient network to photosensitive structure, such as azobenzene, the SMHs display a smart shape alternation upon certain wavelength irradiation (Miyamae et al., 2015; Pan et al., 2016). When the supramolecular ligands are incorporated, the SMHs can response to copper ion (Löwenberg et al., 2017) and silver ion (Löwenberg et al., 2017). Recently, *Liang et al. (2019b)* prepared a SMH by synergetic integration of 2-phenoxyethyl acrylate (PEA) with acrylamide (AAm). The hydrogel possessed strong mechanical strength and by tuning the gel formation at body temperature, the SMH would be a promising tool for transcatheter arterial embolization. *Lu et al. (2015)* utilized two types of DNA networks on the polyacrylamide polymer to form hydrogel. One is duplex DNA which performed as permanent network. The other one can be varied between G quadruplex sensitive to K^+^ and i-motif sensitive to H^+^. When the hydrogel meets its target, K^+^ or low pH, the transient shape may be disturbed to reverse the eternal shape. Of interest, due to the polymer chains’ entanglement and the related fixed distance between two types of networks, after the addition of factor to clear the target, the shape will again fall into the transient shape.

#### Nanocomposite Hydrogels

As the development of nanoscience, in recent years, hydrogel nanocomposite systems, such as magnetic (Abenojar et al., 2018; Jalili et al., 2016), electroconductive (Walker et al., 2019), and quantum dot nanocomposite hydrogels (Javanbakht and Namazi, 2018), are becoming popular for their tailored properties. These hydrogels are composed of polymers in multiphase structure with one phase’s size less than 100 nm to contain various nanomaterials (Bao et al., 2019). They could be programed and applied in biomedical fields, including therapeutic drug delivery, bioimaging, and tissue regeneration engineering. The newly developed nanocomposite hydrogels are summarized in Table 1. *Amini-Fazl et al. (2019)* designed Fe_3_O_4_-based magnetic nanocomposite hydrogel system by loading anticancer drug 5-fluorouracil for colon and rectal administration. Through external magnetic field, the magnetic nanocomposite hydrogel could release drug in target sites.

**TABLE 1:** Recently developed nanocomposite hydrogels.

Classification	Components	Features	Applications
Magnetic	Fe_3_O_4_	Superparamagnetic Low toxicity Biocompatibility Biodegradability	Tissue regenerative Ghuman et al. (2018) Antimicrobial therapy Abenojar et al. (2018) Drug delivery Amini-Fazl et al. (2019)
Conductive	Gold nanoparticle Silver nanoparticles Graphene dots Carbon nanotubes Conductive Polymers	High electrical conductivity High viscoelasticity Electrochemical redox	3D cell cultures Xu Y. et al. (2019) Biosensors Uluturk and Alemdar (2018) Neurogenesis Wu C. et al. (2019) Drug delivery An et al. (2020)
Light emitting	Carbon dots Riboflavin Rhodamine B	Optical tenability High sensitivity Dynamic detection	Tissue regeneration Chichiricco et al. (2018) Drug delivery Javanbakht and Namazi (2018)

The electrically conductive components including gold and silver nanoparticles, graphene, carbon nanotubes, and conductive polymers (polyaniline, polypyrrole, and polythiophenes) are incorporated, doped, or chemically modified in the polymer network to form nanocomposite hydrogel which can be used as scaffolds supporting for cell growth both *in-vivo* and *in-vitro* (Shi et al., 2016; Walker et al., 2019). Xu et al. (2018) formulated smart hydrogel by non-covalent cross-linking PEG–peptide with conductive poly(3,4-ethylenedioxythiophene): polystyrene sulfonate to mimic extracellular matrix structures. The rheology and electrical impedance showed high tenability that can be applied for 3D cell cultures with electric field as the cell differentiation modulator. Graphene quantum dots are promising materials for drug delivery and cellular imaging with excellent properties, including quantum confinement, edge effects, good solubility, various emitting wavelengths, and low toxicity (Javanbakht and Namazi, 2018). *Rakhshaei et al. (2020)* developed a functional biocompatible hydrogel in which the graphene quantum dots acted as crosslinker to link sodium carboxymethyl cellulose (CMC). Due to the outstanding photoluminescent feature for graphene quantum dots and pH-sensitive swelling properties of CMC, the formed hydrogel was capable for bioimaging and low pH-triggered oral drug release.

### Chemical Responsive

#### Glucose Responsive Hydrogels

Glucose is a key biomedical analyte, which plays a crucial role in physiological metabolism. Abnormal glucose levels are associated with diabetes and hypoglycemia. Hence, glucose is closely related to body function, and sustained glucose monitoring promotes the strict control of blood glucose in diabetic (Ma et al., 2018). Especially, Glucose-responsive system plays an important role in self-regulated insulin delivery to treat diabetes (Yin et al., 2019). Recently, *Li et al. (2017)* developed a novel biocompatible glucose-responsive hydrogel loaded with glucose oxidase, catalase, and insulin to deliver insulin for diabetes treatment. In this work, the peptide self-assembles into hydrogel under physiological conditions. Once hypoglycemia happened, adjacent alkaline amino acid side chains are significantly repulsed due to the decreased local pH caused by the enzymatic conversion of glucose into gluconic acid. Then, the subsequent unfolding of individual hairpins leads to disassembly and release of insulin. The blood glucose levels can be well regulated *in vitro* and *in vivo* by the hydrogel. Based on a smart hydrogel system, Wu M et al. 2019) developed a visual detecting approach for glucose sensing, which is assembled by a photosensitive crosslinking hydrogel coupled with a pH-responsive nanogel. The hydrogel system could response quickly sensitively to glucose under physiological condition. Importantly, it is capable to detect glucose visually both *in vitro* and in diabetic mouse models. *Elsherif et al. (2019)* have fabricated the optical fiber probe for continuous glucose monitoring under physiological conditions. The glucose-responsive hydrogel made of (3-(acrylamido)-phenylboronic acid) coupled acrylamide was chemically attached to the silica multimode fiber for glucose monitoring by optical powermeter or even a smartphone. Of note, by substituting the silica fiber by polyethylene glycol diacrylate (PEGDA) hydrogel, the system could still work but with more biocompatibility.

#### pH-Responsive Hydrogels

One of the most outstanding intelligent hydrogels is the pH-responsive hydrogels due to significant variations of pH at different sites under normal and pathological conditions. Several pH-responsive polymers, including DNA, chitosan, and poly (methacrylic acid) (PMAA), have been extensively recommended and used for local controlled drug delivery system. When pH altered following the disease’s occurrence, the hydrogels can trigger disease-controlled drug release. After triggered by pH changing, hydrogels have a gel–sol conversion which is beneficial for drugs encapsulation at gel status and effective frameworks clearance after hydrogel degradation. Until now, several natural polymers like cellulose, hyaluronic acid, guar gum, and chitosan, owing to the presence of abundant ionic groups in their network, have been employed to synthesize pH-responsive hydrogels. Some functional groups like amide, sulfate, phosphate, and carboxylate could be also introduced to alter the electrochemical properties (Khan et al., 2019). Functional DNAs such as triplex structure (Hu et al., 2015; Lu et al., 2018) and i-motif sequence (Cheng et al., 2009; Xu et al., 2017) are good candidates of another category for the construction of pH-sensitive hydrogels. *Lu et al. (2018)* designed a pure DNA hydrogel based on triplex DNA structures with protonated cytosine–guanine–cytosine (C-G•C+) and thymine–adenine–thymine (T-A•T) to set the pH transition point to 5.0 and 7.0, respectively. The highly tunable pH responsiveness made it attractive in biomedical applications.

### Biochemically Responsive

#### Enzyme-Sensitive Hydrogels

Hydrogels in the biomedical application are usually exposed to enzymes in the tissue. Recently, hydrogels have been focused on their specific enzyme responsive property, especially for those disease-related enzymes like matrix metalloproteinases (MMPs). MMPs are upregulated in varieties of diseases, attracting attention on the design of MMP-responsive hydrogel for drug delivery (Bae and Kurisawa, 2016). For instance, *Zhao Z. et al. (2020)* used an injectable MMPs sensitive hydrogel that payload temozolomide (TMZ) and O^6^-benzylamine (BG) for the clearance of residual TMZ-resistant gliomas after surgery. When the concentration of MMPs enzymes is high, hydrogels could release the TMZ and BG simultaneously when TMZ kills the residual glioma cell under the guarantee by BG. The result is demonstrated to be good in localized drug codelivery for residual glioma treatment. The hydrogel embedded in the arthritic joint with lots of therapeutic agents and enzyme sensing agents will disassemble when enzyme appears in the inflammatory microenvironments. Instead of providing prolonged drug release, enzyme-sensitive hydrogels are capable to supply drug one time when necessary, thereby improving therapeutic efficacy (Joshi et al., 2018).

#### Reactive Oxygen Species-Sensitive Hydrogels

ROS, such as superoxide (O_2_^−^), singlet oxygen (^1^O_2_), hydroxyl radical (·OH), peroxynitrite (ONOO^−^), and hypochlorite (OCl^−^), play important roles in biological process. However, abnormal metabolism elevates ROS level significantly in many diseases (Saravanakumar et al., 2017; Hampton, 2018). For the ROS-responsive hydrogels, they can sense local environment oxidative stress, regulate cell behavior, accelerate drug release, and finally remove redundant ROS. Commonly, the ROS-sensitive polymers can be synthesized *via* polymerization of or post polymerization modification with ROS-responsive moieties. Thioether, selenium, tellurium, thioketals, phenylboronic acids/esters, and oxalate-containing structures are included. There are mainly two strategies to engineer ROS-sensitive hydrogels (Ye et al., 2019). One is to merge ROS-responsive moieties into the block polymer backbones. For instance, *Zhu et al. (2018)* incorporated ROS scavenging groups 4-amino-2,2,6,6-tetramethylpiperidine-1-oxyl (4-amino-TEMPO) to the hydrogel scaffold to protect tissue from free radical hurting locally. The other strategy is to incorporate ROS-sensitive side chains into hydrogels. *Martin et al. (2020)* designed ROS-degradable PEG hydrogel cross-linked with ROS-degradable poly (thioketal) by thiol–maleimide chemistry. The hydrogel’s degradation rate specifically correlates with the cell-generated ROS, and the antioxidative function makes it suitable for encapsulating mesenchymal stem cells with higher cell viability. *Yu S. et al. (2018)* designed a biodegradable injectable hydrogel composed of PEG, ROS-responsive l-methionine (Me), and dextro-1-methyl tryptophan (D-1MT), loading with anti-programed cell death-ligand 1 (aPD-L1) effectively. The encapsulated aPD-L1 and D-1MT could be released from the intelligent hydrogels slowly according to the trigger of environmental ROS that will result in the reduction of ROS level and enhanced antitumor efficacy.

## Conclusions and Future Perspectives

In this mini review, we supplied a comprehensive perspective over the rational hydrogel design principle from the molecular constitutional basis to function-guided tailoring in the biomedical field.

The unique properties of hydrogels make it a wonderful platform for biomedical applications, although there are still varieties of obstacles to overcome. First, hydrogels composed of different molecules possess merits of their own; the natural polymer is biocompatible with low toxicity but weak, while the synthetic polymer is often cell toxic but strong. How to mingle the advantages together is a hard question. The mechanism of multipolymer network and shape memory hydrogel can provide some suggestions for further advancing. Second, the contemporary hydrogels are mainly designed for a single purpose (*e.g.,* different stimuli triggered drug delivery), but in practical biomedical applications, more work need to be done. We demand a unity to monitor, to sense, and to stimulate with the feedback loop system to smartly control the drug release rate, or even release the reverse drug to terminate the therapy when disease changes. The constructure of such unity can draw experience from cascaded assembly or cascaded enzymatic reaction. Third, as the emerging of a lot of new hydrogels as the development of nanoscience and pharmacy, by incorporation with functional nanomaterial and emerging functional peptide and DNA, the hydrogel will move beyond the present stimuli target, even alter the present hydrogel designed mechanism. As for the high specificity of functional peptide and DNA, we foresee that the hydrogel could be designed specifically to personalized individuals.

## Author Contributions

YZ contributed to the literature collection, draft writing, and manuscript preparation; YH contributed to the conception of the review outline and manuscript preparation. Both authors provided critical feedback.

## Funding

The work is supported by grants from the National Natural Science Foundation of China (No 21705074), Xiamen Youth Innovation Fund Project (3502Z20206064), Education Scientific Research Project for Young and Middle-aged Teachers in Fujian Province (JAT190989).

## Conflict of Interest

The authors declare that the research was conducted in the absence of any commercial or financial relationships that could be construed as a potential conflict of interest.
